# Functional characterization of two enhancers located downstream *FOXP2*

**DOI:** 10.1186/s12881-019-0810-2

**Published:** 2019-05-02

**Authors:** Raúl Torres-Ruiz, Antonio Benítez-Burraco, Marta Martínez-Lage, Sandra Rodríguez-Perales, Paloma García-Bellido

**Affiliations:** 1 Molecular Cytogenetics Group, Centro Nacional Investigaciones Oncológicas (CNIO), Madrid, Spain; 2 Department of Spanish, Linguistics, and Theory of Literature (Linguistics), University of Seville, Seville, Spain; 3 Faculty of Modern Languages, University of Oxford, Oxford, UK; 4 Faculty of Linguistics, Philology and Phonetics, University of Oxford, Oxford, UK

**Keywords:** *FOXP2*, *MDFIC*, Speech and language impairment, Spanish, CRISPR-genome editing, Functional enhancers, Chromosomal rearrangement

## Abstract

**Background:**

Mutations in the coding region of *FOXP2* are known to cause speech and language impairment. However, it is not clear how dysregulation of the gene contributes to language deficit. Interestingly, microdeletions of the region downstream the gene have been associated with cognitive deficits.

**Methods:**

Here, we investigate changes in *FOXP2* expression in the SK-N-MC neuroblastoma human cell line after deletion by CRISPR-Cas9 of two enhancers located downstream of the gene.

**Results:**

Deletion of any of these two functional enhancers downregulates *FOXP2*, but also upregulates the closest 3′ gene *MDFIC*. Because this effect is not statistically significant in a HEK 293 cell line, derived from the human kidney, both enhancers might confer a tissue specific regulation to both genes. We have also found that the deletion of any of these enhancers downregulates six well-known FOXP2 target genes in the SK-N-MC cell line.

**Conclusions:**

We expect these findings contribute to a deeper understanding of how *FOXP2* and *MDFIC* are regulated to pace neuronal development supporting cognition, speech and language.

**Electronic supplementary material:**

The online version of this article (10.1186/s12881-019-0810-2) contains supplementary material, which is available to authorized users.

## Background

Mutations in the coding region of the *FOXP2* gene, encoding a forkhead transcription factor, are known to cause speech and language impairment [1–6]. Polymorphisms of the gene have also been associated with schizophrenia [7](Tolosa et al., 2010) and frontotemporal lobar degeneration [8]. *FOXP2* has been hypothesised to regulate the development and function of brain areas involved in language processing [1, 9, 10], because of its known role in neurogenesis, neuron differentiation, and neuron migration in the developing telencephalon of mice [11–13]. Pathogenic mutations in humans have proven to impair auditory-motor association learning when mimicked in mice [14]. Nonetheless, the exact role of *FOXP2* in normal development of the human brain and cognition is unknown. Common variants of the gene do not contribute appreciably to individual differences in language development [15], nor in brain structure [16], although a *FOXP2* polymorphism has been recently associated with enhanced procedural learning of non-native speech sound categories [17]. Less is known about how the expression of the gene is modulated. The promoter of *FOXP2* contains four transcription start sites [18], with multiple alternative splicing sites [19]. *FOXP2* also contains six ultraconserved regions in its introns [18], as well as six predicted enhancers for lef1 [20], a transcription factor that drives expression of *foxP2* in the central nervous system during zebrafish embryogenesis [21]. Interestingly, several microRNAs bind the 3’UTR of the *FOXP2* gene and regulate the expression of the gene [22].

Pathogenic microdeletions involving the *FOXP2-MDFIC* region resulting in language or cognitive impairment have been reported (Additional file 1: Figure S1). Most microdeletions have removed partially the 3′ end of the *FOXP2* gene, likely resulting in altered expression levels of *FOXP2* and/or shorter aberrant FOXP2 proteins. It has been recently reported on a young female, harbouring a de novo balanced complex rearrangement involving one copy of chromosomes 7 and 11, who presents with a severe developmental expressive and receptive speech and language impairment in two language modalities: Castilian Spanish and Valencian [23]. The rearrangement of this clinical case does not interrupt any protein-coding sequence in derivative chromosomes 7 or 11, and no other protein-coding gene, close to the regions interrupted by the rearrangement except *FOXP2,* is considered to be a strongly suspected candidate for the phenotype observed [23]. Although the *FOXP2* coding region is intact, the breakpoint in 7q31.1 is located 205.5 kb downstream the 3′ end of *FOXP2*, suggesting that it might have affected some regulatory region of the gene. Based on FISH results from the RP11-243D16 BAC, it has been proven that in this proband *FOXP2* is de novo rearranged to derivative chromosome 11p [23]. Becker and collaborators [24] identified and characterized in a luciferase assay a functional enhancer located 2.5 kb downstream the breakpoint, here FOXP2-E^distal^, and hypothesized that separating this putative regulatory element from the coding region of FOXP2 would have contributed to the observed language phenotype by disturbing *FOXP2* gene expression. The putative gene-specific regulatory role of this characterised element needed to be tested.

The development of nuclease mediated genome editing tools, specially, of those based on clustering regularly interspaced short palindromic repeats (CRISPR) [25–27], has emerged as an efficient way of inducing targeted chromosomal deletions and an accurate method to validate the functionality of enhancers [28, 29]. Here we report a detailed functional study of the intergenic region between *FOXP2* and *MDFIC* genes. It has been found that this region contains, apart from the enhancer found in Becker’s report [24], another functional enhancer, here FOXP2-E ^proximal^. We performed a targeted deletion of one regulatory element per cell by CRISPR-Cas9 and found that whereas *FOXP2’s* mRNA and protein levels decreased, *MDFIC’s* mRNA and protein levels increased. We hypothesise that the breakpoint in chromosome 7q31.1 of the proband [23] may have disrupted this intergenic regulatory region causing, anomalously, *FOXP2* to be downregulated and *MDFIC* upregulated. Changes in the expression levels of these two adjacent protein-coding genes in particular brain circuits during development may have led to the observed language deficits in the proband. We expect these findings contribute to a better understanding of how *FOXP2* is regulated.

## Methods

### Cell culture and electroporation

HEK293 (CRL-1573, ATCC, USA) and SK-N-MC (HTB-10, ATCC, USA) cells were cultured in Dulbecco’s modified Eagle’s medium (DMEM) (Lonza) using standard conditions: DMEM medium was supplemented with 10% foetal bovine serum (FBS) (Life Tech), 1% GlutaMAX (Life Tech) and 100 units/ml penicillin/streptomycin (Life Tech). Cells were cultured at 37 °C in a humidified atmosphere of 5% CO_2_ + 20% O_2_. Cells were passed when they reached at 80% of confluence.

SK-N-MC and HEK293 cells were electroporated with 2μg of either pLV-U6^#x^-C9G, pLV-U6^#x^H1^#y^-C9G or with an empty plasmid. For electroporation, we used the Neon Transfection System (Life Technologies) as previously described [30]. The manufacturer’s protocols for HEK293 and SK-N-MC cells were modified as follows. Cells were trypsinized and resuspended in R solution (Life Technologies). For SK-N-MC 10-μl tips were used to electroporate 2.5 × 10^5^ cells with a single 50-ms pulse of 900 V. For HEK293 cells, 4 × 10^5^ cells were electroporated with 10-μl tips using three 10-ms pulses of 1245 V. After electroporation, cells were seeded in a 24-well plate containing pre-warmed medium. When required, cells were sorted 72 h post-transfection.

### sgRNA design and construction of single- and double-guide Cas9-encoding plasmids

The CRISPR sgRNAs were designed using the http://crispr.mit.edu/ and https://benchling.com/ online tools that were also used to evaluate their off-target scores. The parental pLV-U^#x^-C9G and pLV-U6^#x^H1^#y^-C9G vector has been described elsewhere [31]. Eight gBlocks gene fragments (IDT) were synthesized to clone the sgRNAs separately or in the following combinations sgEp#1-sgEp#3, sgEp#1-sgEp#4, sgEp#2-sgEp#3, sgEp#2-sgEp#4, sgEd#1-sgEd#3, sgEd#1-sgEd#4, sgEd#2-sgEd#3 or sgEd#2-sgEd#4 (Additional file 7: Table S1), flanking the FOXP2-E^proximal^ and FOXP2-E^distal^ enhancer regions in the backbone vector using BsrGI and SpeI target sites.

### TIDE assay

72 h post-electroporation genomic DNA was extracted using DNeasy blood and tissue kit (QIAGEN) following manufacturer’s instructions. 300–500 bp PCR amplicons spanning the sgRNA genomic target sites were generated using the primers shown in Additional file 7: Table S1. PCR products were purified with QIAquick PCR purification kit (QIAGEN) and Sanger-sequenced using both PCR primers and each sequence chromatogram was analysed with the online TIDE software available at http:// tide.nki.nl. Analyses were performed using a reference sequence from a control cell sample electroporated with the pLV-U6H1-C9G empty vector. Parameters were set to the default maximum indel size of 10 nucleotides and the decomposition window to cover the largest possible window with high quality traces. All TIDE analyses below the detection sensitivity of 3.5% were considered as non-significant targeted [32, 33].

### Flow cytometry and cell sorting

72 h after electroporation, cells were trypsinized, counted, and resuspended at the concentration of 3-5 × 10^6^ cells/ml in an appropriate volume of sorting buffer (phosphate-buffered saline (PBS)/0.5% bovine serum albumin (BSA)/2 mM EDTA and 100 units/ml penicillin/streptomycin (Life Technologies)) for flow cytometry analysis. Immediately before cell sorting, samples were filtered through a 70-μm filter (Miltenyi Biotec) to remove any clumps or aggregates. Sorting was performed in sterile conditions throughout the experiments in the sorting buffer described above recovering at least 0.5 × 10^6^ cells per experimental condition. Cell sorting was carried out in a Synergy 2 L instrument (Sony Biotechnology Inc.); flow cytometry was performed in a BD LSR Fortessa analyzer (BD Biosciences) and FACSDiva software was used. Proper electronic gates of side scatter and forward scatter parameters were set in order to exclude cell debris and dead cells. The SK-N-MC or HEK293 GFP negative control cells were analysed in order to assess the minimal fluorescein isothiocyanate baseline. Sorting was performed in sterile conditions throughout the experiment. Sorted cells were seeded individually per well in a 96 well-plate containing DMEM supplemented medium.

### Genomic DNA extraction and PCR analysis

Standard procedures were used for genomic DNA extraction 72 h post-electroporation [31]. Briefly, 10 × 10^6^ cells were lysed in 100 mM NaCl, Tris (pH 8.0) 50 mM, EDTA 100 mM, and 1% SDS followed by overnight digestion with 0.5 mg/ml of proteinase K (Roche Diagnostics) at 56 °C. Afterward, the DNA was cleaned by NaCl precipitation, precipitated with isopropanol and resuspended in 1xTE buffer. NanoDrop ND 1000 Spectrophotometer (NanoDrop Technologies) was used to quantified DNA.

For deletion analysis and homozygous versus heterozygous representation standard PCR and three primer strategy analysis were performed in a Veriti 96-well Thermal Cycler (Applied Biosystems) under the following conditions: 95 °C denaturation for 1 min followed by 30 cycles of denaturation at 94 °C, annealing at 62.5 °C, extension at 72 °C, and a final extension at 72 °C. Primers used are described in Additional file 7: Table S1.

### RNA extraction and qRT-PCR

Trizol (Sigma-Aldrich) protocol followed by RNase-free DNAse (Roche Applied Science) treatment was used to extract total RNA from cell cultures. 500 ng of total RNA was used for cDNA synthesis using the Superscript III First Strand cDNA Synthesis Kit (Life Tech). Quantitative real-time PCR was performed on an ABI Prism 7900 HT Detection System (Applied Biosystems) with SDS 2.1 software. The reaction mix contained TaqMan master mix (Thermo Fisher) and 0.3 μM of each primer (Additional file 7: Table S1). Cycling conditions were 50 °C for 2 min and 95 °C for 3 min, followed by 40 cycles at 95 °C for 15 s and 58 °C for 45 s, and, finally, 95 °C for 15 s, 60 °C for 20 s, and 95 °C for 15 s.

PCR was performed in 96-well plate microtest plates. In all experiments, hGAPDH (internal reference control) was used to normalize mRNA amounts to the total amount of cDNA. Each sample was determined in triplicate, and three independent samples were analysed for each experimental assay.

### Western blot

Proteins were extracted by standard procedures as previously described [34] in the presence of Complete Protease Inhibitor Cocktail Tables (Roche Applied Science). Control cells are SK-N-MC electroporated with the pLV-U6^#^H1^#^-C9G plasmid. Proteins were wet-transferred with TransFi (Invitrogen; Life Technologies) to polyvinyl difluoride (PVDF) membranes (Hybond-P, Amersham Biosciences) for 90 min. The protein-bound membranes were blocked with non-fat dry milk in Tris-buffered saline with Tween-20 at room-temperature and then incubated overnight at 4 °C with monoclonal mouse anti-human FOXP2 or MDFIC antibodies (1/1000 or 1/500; BD Pharmigen) or with rabbit anti-human GAPDH antibody (1/500; AbCam). After several PBS-T washes the membranes were incubated for 1 h at room temperature with secondary antibodies horseradish peroxidase (HRP)-conjugated with goat anti-mouse (1/1000) and goat anti-Rabbit (1/500; Dako, Barcelona, Spain) diluted in PBS-T. After several PBS-T washes the membranes were developed with enhanced chemiluminiscence (ECL) (GE Healthcare). The ECL signals were visualized on X-ray films. The FOXP2 and MDFIC proteins were normalized with the GAPDH internal control in the same lane.

### Statistical analysis

Data from three independent experiments were analysed by two-tailed Student’s t-test using Excel (Microsoft). All data are expressed as means ± s.e.m. *P* < 0.05 was considered statistically significant. * *p* < 0.05; ** *p* < 0.01; *** *p* < 0.001; and **** *p* < 0.0001.

## Results

### In silico search of enhancer regions

**Fig. 1:**
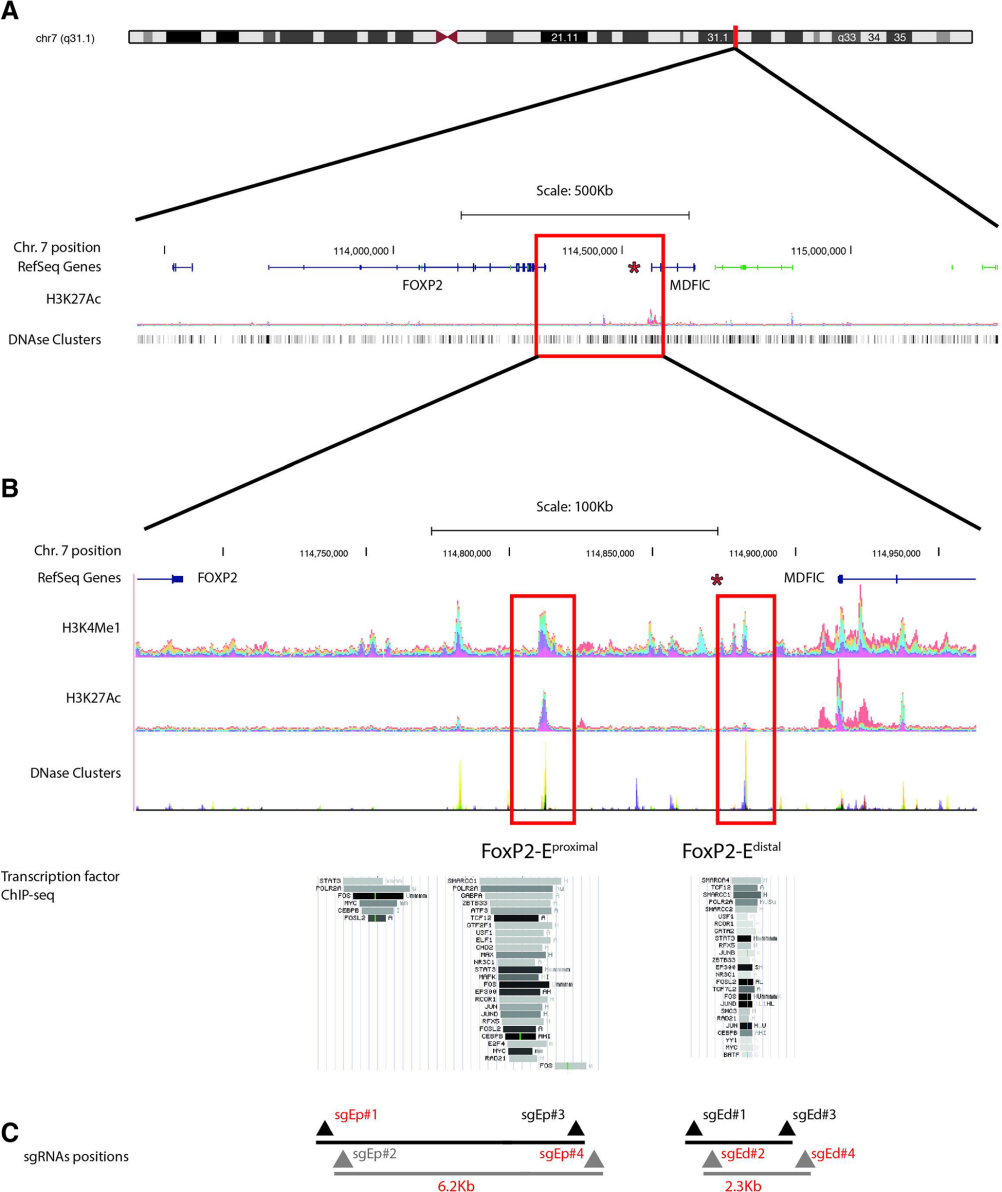
Identification of enhancer regions downstream *FOXP2* and upstream *MDFIC*. **a**. Genomic location of human *FOXP2* and *MDFIC* genes (GRCh38/hg38). The red asterisk shows the intergenic position of the 7q31.1 breakpoint in the proband harbouring a genomic complex rearrangement of intact *FOXP2* and with severe expressive and receptive speech and language impairment. **b**. Detailed view of an ENCODE UCSC genome-browser snapshot showing bar graphs with a detailed representation of the locations of H3K4Me1 and H3K27Ac histone marks and DNA clusters in human cell lines. The squared regions in red show the locations of FOXP2-E^proximal^ and FOXP2-E^distal^. The red asterisk shows the position of the 7q breakpoint in the proband harbouring a genomic complex rearrangement and with severe expressive and receptive speech and language impairment. The transcription factor track shows transcription factor binding sites obtained from a collection of ChIP-seq experiments. A grey horizontal box (32 for FOXP2-E ^proximal^ and 25 for FOXP2-E^distal^) encloses each transcription factor cluster, with the darkness of the box being proportional to the maximum signal strength. The transcription factor name is shown to the left of each box. As seen in the figure the majority of transcription factors bind to both enhancers. **c**. Schematic representation of the location of the four sgRNA pairs flanking the 6.2 kb region including FOXP2-E^proximal^ and the 2.3 kb region including FOXP2-E^distal^. sgRNAs with the highest cleavage efficiencies are labelled in red

We first hypothesised that the breakpoint in 7q31.1 (chr7:114,888,284 hg38 equivalent to 114,539,340 hg19) affected the expression of *FOXP2* by physically disrupting a regulatory region downstream the coding region of the gene containing cis-acting distant elements with an enhancer role (Fig. 1a). Accordingly, we used the Encyclopedia of DNA Elements (ENCODE, https://genome.ucsc.edu/ENCODE/) to perform in silico searching for putative enhancers in the intergenic region between *FOXP2* and *MDFIC*. We looked for the following hallmarks from ENCODE: DNase I hypersensitive sites, presence of histones with specific post-translational modifications (in particular, histone H3, lysine 4 monomethylation (H3K4Me1) and H3 lysine 27 acetylation (H3K27Ac)), and regions recruiting co-activators and co-repressors as revealed by chromatin immunoprecipitation followed by deep sequencing (ChIP-seq) (Fig. 1b). The ENCODE data, derived from a large collection of different cell types, including hippocampal cells, cerebellar cells, and cells from the spinal cord, showed two putative enhancers located at 120 kb and 208 kb downstream the stop codon of *FOXP2*, respectively (Fig. 1b). These putative enhancers (referred as FOXP2-E^proximal^ and FOXP2-E^distal^) span 6263 bp (chr7:114,817,431-114,823,694 hg38 equivalent to 114,456,873-114,463,136 hg19) and 2313 bp (chr7:114,900,989-114,903,302 hg38 equivalent to 114,541,370-114,543,683 hg19), respectively. FOXP2-E^distal^ is the one previously validated by luciferase assay [24]; FOXP2-E^proximal^ is a new putative regulatory element.

### CRISPR-Cas9 deletion of FOXP2-E^proximal^ and FOXP2-E^distal^

**Fig. 2:**
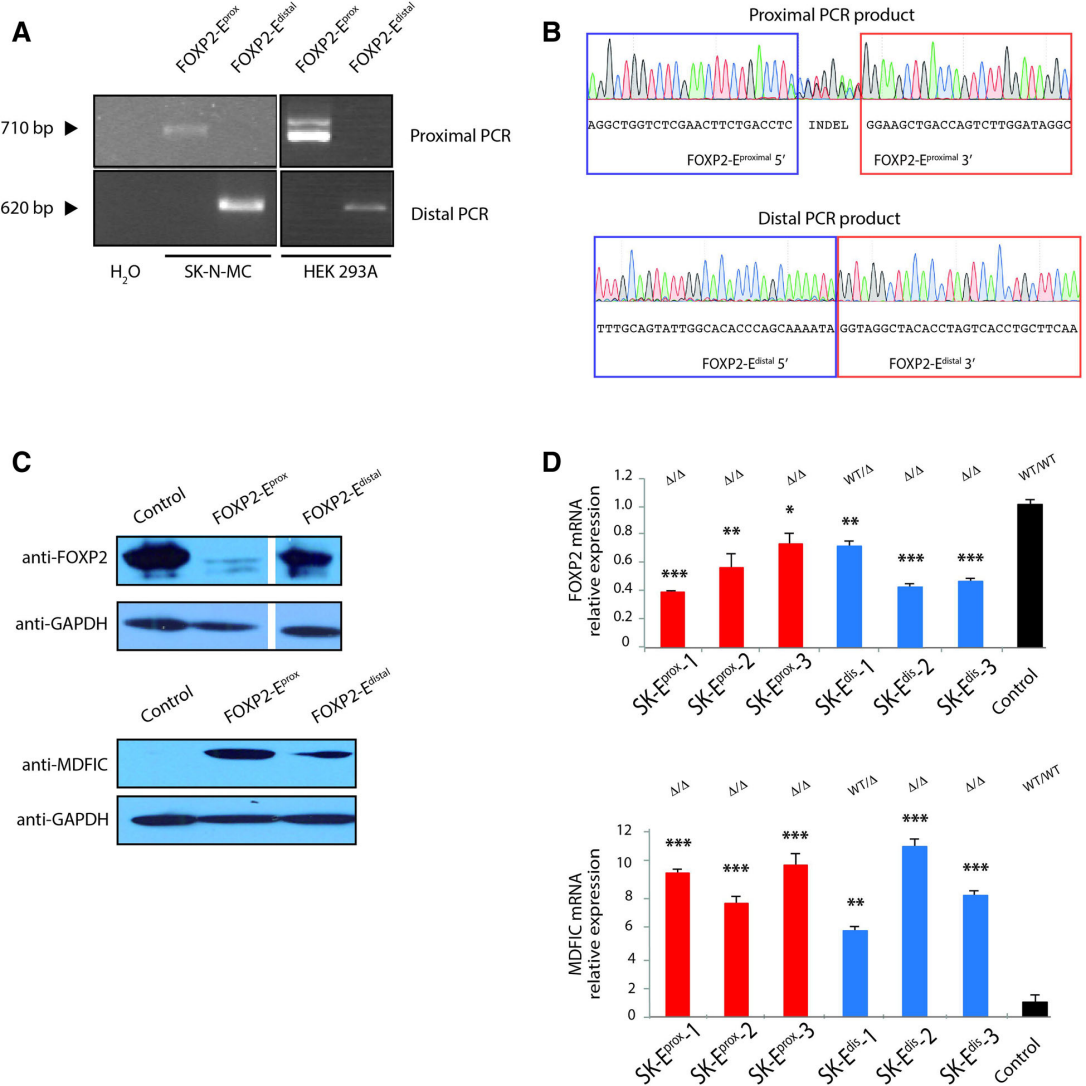
Molecular characterization of FOXP2-E^proximal^ and FOXP2-E^distal^. **a**. PCR analysis. Two oligos flanking the deleted regions were used to amplify the genomic DNA from two FOXP2-E^proximal^ and two FOXP2-E^distal^ deleted representative SK-N-MC and HEK293 cells. Black triangles show the size of the PCR products. **b**. Representative Sanger sequencing chromatogram showing the sequences of the junctions of the FOXP2-E^proximal^ (top) and FOXP2-E^distal^ (bottom) genomic deleted regions in a SK-N-MC cells. **c.** Western blot analysis of cell lysates of SK-N-MC with FOXP2-E^proximal^ deleted, with FOXP2-E^distal^ deleted, and of SK-N-MC control cells electroporated with pLV-U6^#^H1^#^-C9G plasmid for FOXP2 (top) or MDFIC (bottom) proteins analysis. **d**. qRT-PCR analysis in triplicate as technical replicates of six SK-N-MC cell clones with FOXP2-E^proximal^ or FOXP2-E^distal^ deletions, control cells are SK-N-MC cells electroporated with the pLV-U6^#^H1^#^-C9G plasmid. Both deleted and control values are normalized to those of the internal reference gene hGUSB. Levels of expression of *FOXP2* (up) and *MDFIC* (down) are represented by the fold change relative to that of empty vector control cell line, which was normalized to 1. Data from three or more independent experiments were analysed by two-tailed unpaired t-test. NS, non-significant; * *p* < 0.05; ** *p* < 0.01; *** *p* < 0.001; and **** *p* < 0.0001

Deletion of an enhancer motif provides direct evidence for enhancer activity, CRISPR-Cas9 being the gold standard to study its role in the regulation of endogenous gene transcription. To investigate the regulatory role of FOXP2-E^proximal^ and FOXP2-E^distal^, we specifically deleted the entire predicted sequence of one or another of the two putative enhancers in a cell line of neuroectodermal human origin. Previous attempts to measure *FOXP2* expression in primary skin fibroblasts from the proband failed because mRNA levels were too low for significant statistical analysis [23]. Expression levels of *MDFIC* mRNA*,* obtained by Affymetrix gene expression arrays from primary skin fibroblast from the proband, were found to be higher than those of FOXP2, but were not independently validated ([23], Additional file 5: Table S5). SK-N-MC neuroblastoma cells derive from the supraorbital area and express *FOXP2* and *MDFIC* constitutively. To generate a specific deletion we relied on a CRISPR-Cas9 genome editing approach. In our strategy, two independent pairs of sgRNAs are used to target the flanking regions of either FOXP2-E^proximal^ or FOXP2-E^distal^ to induce repair of the resultant two double strand breaks (DSBs) by non-homologous end-joining (NHEJ) with deletion of the intervening segment (Fig. 1c). First, two sgRNAs were designed per flanking region (eight in total, Additional file 7: Table S1) and cloned them separately in the pLV-U6^#x^-C9G vector [31] in order to analyse their specificity and efficiency of cleavage. SK-N-MC cells were electroporated with one of the pLV-U6^#x^-C9G single sgRNA expression vectors to precisely determine the on-target (efficiency) and off-target (specificity) DNA modification frequencies in the pooled edited cell population by tracking of indels by decomposition (TIDE) assay [32]. As shown in Additional file 2: Figure S2 the on-target cleavage efficiencies, measured by INDEL frequency, ranged from 3.9 to 17.1% whereas no significant off-target activity was observed at the predicted top off-target sites (Additional file 3: Figure S3). The four flanking sgRNAs, two for each deletion, with the highest cleavage efficiencies were selected and cloned by pairs in the pLV-U6^#x^H1^#y^-C9G [31] in order to couple its expression with Cas9 nuclease and GFP reporter generating the pLV-U6^#1^H1^#4^-C9G-E^proximal^ and pLV-U6^#2^H1^#4^-C9G- E^distal^ vectors. 72 h post-nucleofection with the double-guide or empty pLV-U6^#^H1^#^-C9G control vector PCR analyses of the SK-N-MC bulk cell populations confirmed the deletion of the 6.2 kb or the 2.3 kb expected fragments (Figs. 1c and 2a-left panel). Sanger sequence analysis confirmed the presence of the deletions (Fig. 2b). We then generated five FOXP2-E^proximal^ and three FOXP2-E^distal^ deleted clonal cell lines, by sorting GFP positive cells into 96-well plates and allowing for single cell colony expansion. A three primer PCR approach confirmed higher rates of homozygous compared to heterozygous clones. The five FOXP2-E^proximal^ deleted clones harboured a homozygous deletion, whereas only one of three FOXP2-E^distal^ deleted clones harboured a heterozygous deletion (Additional file 4: Figure S4 bottom panels). PCR analysis revealed that two out of the five FOXP2-E^proximal^ clonal cell lines having harboured the deletion also contained small insertion/deletions produced at CRISPR recognition sites and consequently were excluded from our study (Data not shown). Interestingly, clones with homozygous deletion could readily be isolated, but only one heterozygous clone could be obtained. This is potentially due to the efficiency of the Cas9 to perform genome engineering which results mostly in bi-allelic edition [28, 35–38]. SK-E^prox^-1, SK-E^prox^-2 and SK-E^prox^-3 homozygous clones, SK-E^dis^-1 heterozygous and SK-E^dis^-2 and SK-E^dis^-3 homozygous clones were selected for further analysis.

To test whether the FOXP2-E^proximal^ and/or FOXP2-E^distal^ regions play a functional role in other cell types, we deleted one predicted enhancer per non-neuronal HEK293 cell. HEK293 cells have been used previously to show functionality of the FOXP2-E^distal^ by a luciferase assay [24]. These easy-to-transfect cells, have been extensively used as a quick and straightforward system to characterize gene function and enhancer prediction [39–41]. HEK293 cells were nucleofected with either pLV-U6^#1^H1^#4^-C9G-E^proximal^, pLV-U6^#2^H1^#4^-C9G-E^distal^ vectors or with an empty pLV-U6^#^H1^#^-C9G control plasmid. 72 h post-transfection PCR analysis revealed targeted deletion of the 6.2 kb or the 2.3 kb regions, which contain the entire sequence of FOXP2-E^proximal^ or FOXP2-E^distal^, respectively (Fig. 2a right panel). Accordingly, we were able to generate 8 homozygous and 6 heterozygous clonal FOXP2-E^proximal^; and 15 homozygous FOXP2-E^distal^ deleted cell lines by sorting GFP positive cells and single cell colony expansion (Additional file 4: Figure S4 upper panels). PCR analysis confirmed the deletion of either the FOXP2-E^proximal^ or the FOXP2-E^distal^ elements (Fig. 2a-right panel). A three-primer PCR approach was used to analyse the homo or heterozygous deletion status (Additional file 4: Figure S4 upper panels) and the HEK-E^prox^-1 homozygous, HEK-E^prox^-2 heterozygous and HEK-E^prox^-3 heterozygous, HEK-E^dis^-1, HEK-E^dis^-2 and HEK-E^dis^-3 homozygous clones were selected for further analysis.

### *FOXP2* and *MDFIC* expression analyses

We next aimed to characterize in more detail the regulatory expression pattern of FOXP2-E^proximal^ and FOXP2-E^distal^. Since both putative enhancers are located in an intergenic region, we aimed at characterizing that each of them is functional with respect to their flanking genes, *FOXP2* or *MDFIC*. We used Western blot analysis to test the amount of FOXP2 and MDFIC proteins in the SK-N-MC pooled cell clones harbouring either the FOXP2-E^proximal^ or FOXP2-E^distal^ deletions, and also in control cells electroporated with the pLV-U6^#^H1^#^-C9G empty plasmid (Fig. 2c). The deletion of FOXP2-E^proximal^ or FOXP2-E^distal^ was found to reduce the amount of the FOXP2 protein (Fig. 2c top) and to increase the amount of MDFIC (Fig. 2c bottom). A decreasing FOXP2 protein effect was found to be more pronounced in the SKN-M-C cells with a FOXP2-E ^proximal^ deletion than with a FOXP2-E ^distal^ removal.

We used qRT-PCR to determine the amount of FOXP2 mRNA in the SK-N-MC cells harbouring either FOXP2-E^proximal^ or FOXP2-E^distal^ deletions, or a control empty pLV-U6^#^H1^#^-C9G line with a wild type genotype. The analysis of three independent homozygous clones showed a significant reduction (up to 2 fold change) in the mRNA expression of FOXP2 compared to that of the control cells when FOXP2-E^proximal^ was deleted (Fig. 2d, up). Likewise, FOXP2 mRNA expression was decreased (up to 2 fold change) in three SK-N-MC clones when FOXP2-E^distal^ was deleted either in homozygous or in heterozygous clones (Fig. 2d up). We then measured the expression levels of MDFIC in SK-N-MC clones after the deletion of each enhancer. As shown in Fig. 2d-down, the expression of MDFIC was significantly increased when either FOXP2-E^proximal^ or FOXP2-E^distal^ were deleted (up to 9.5 and 11 fold change, respectively).

To further investigate the neuronal specificity of FOXP2-E^proximal^ or the FOXP2-E^distal^ enhancers, we replicated the experiment in randomly selected control HEK293 cell clones harbouring either the FOXP2-E^proximal^ or the FOXP2-E^distal^ deletions or in a control cell line electroporated with the pLV-U6^#^H1^#^-C9G vectors. The engineered HEK293 cells do not exhibit significant alteration of the FOXP2 or MDFIC mRNA expression, compared to the parental HEK293 cells (Additional file 5: Figure S5). We noted that SK-N-MC cells are derived from a neurologic origin whereas HEK293 cells are derived from the kidney. Therefore, MDFIC and FOXP2 regulation of expression and post-transcriptional processes are likely to be different in the two cell types; as expected the changes in the expression of both genes in the SK-N-MC and HEK293 cells upon deletion of FOXP2-E^proximal^ and FOXP2-E^distal^ were different. While in a SK-N-MC cell line deletion of each enhancer has a statistically significant effect by decreasing mRNA transcription levels of *FOXP2* and increasing the levels of *MDFIC*, this effect is of no statistical significance in a HEK 293 cell line (Fig. 2d and Additional file 5: Figure S5). These results indicate that the interacting promoters are, potentially, significantly co-expressed in a tissue-specific manner.

**Fig. 3:**
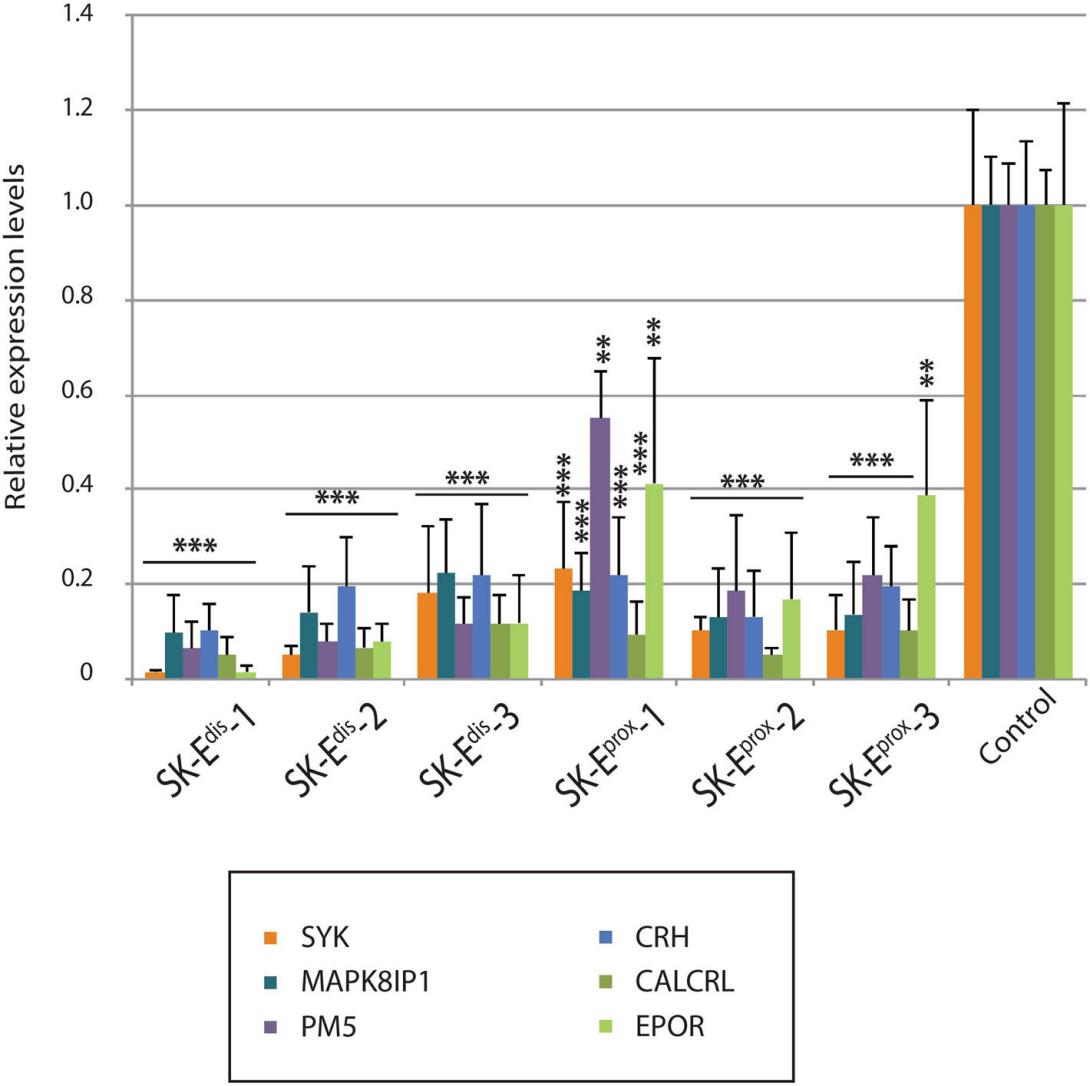
Graphical representation of qRT–PCR analysis of SYK, MAPK8IP1, PM5, CRH, CALCRL and EPOR FOXP2 target genes in SK-N-MC FOXP2-E^proximal^ or FOXP2-E^distal^ enhancers deleted cells and control SK-N-MC cell electroporated with the pLV-U6^#x^H1^#y^-C9G empty vector. *n* = 3. mean ± s.e.m. *P = 0.01–0.05, **P = 0.001–0.01, ***P = 0.001–0.0001, unpaired two-tailed Student’s t-test

Knowing that FOXP2 is a transcription factor that leads to significant changes in the transcription of specific target genes, we tested whether the downregulation of FOXP2 expression after FOXP2-E^proximal^ or FOXP2-E^distal^ was coupled with alterations in the expression level of six well-known FOXP2 target genes: *CALCRL*, *CRH*, *EPOR, MAPK8IP1, PM5,* and *SYK* [42, 43]. Cells transfected with empty pLV-U6^#^H1^#^-C9G plasmid were used as a baseline control for comparison. Real-time quantitative RT-PCR (qRT-PCR) on total RNA extracted from the six SK-N-MC cellular clones described above demonstrated that the six FOXP2 target genes have a significant downregulation in mRNA expression (Fig. 3). These results reinforce the view that each, FOXP2-E^proximal^ and FOXP2-E^distal^, significantly regulates the expression of *FOXP2* and that of six FOXP2 target genes in a human neuronal cell line.

## Discussion

In this paper we have characterised in detail the role of two functional regulatory elements located downstream *FOXP2* employing a CRISPR-Cas9 approach in a neuronal cell line. ENCODE Encyclopedia data suggested that two putative enhancers are localized in the intergenic region between *FOXP2* and *MDFIC,* located at 120 kb and 208 kb downstream the stop codon of *FOXP2*. FOXP2-E^distal^ had been previously found to be functional in an overexpression luciferase assay [24], whereas FOXP2-E^proximal^ was previously uncharacterized. Using CRISPR-Cas9 approach we could readily isolate clones with homozygous deletion, but, interestingly, only a very low number of heterozygous clones could be isolated, possibly due to the efficiency of Cas9 nuclease [44]. We have now proved that if FOXP2-E^distal^ is deleted, *FOXP2* becomes downregulated and the levels of FOXP2 protein are reduced in the SK-N-MC neuroblastoma cells. We have further proved that, if FOXP2-E^distal^ is deleted, it also affects the expression of the adjacent downstream gene, *MDFIC*, by increasing its mRNA and protein levels. We have found that FOXP2-E ^proximal^ deletion, as that of FOXP2-E^distal^, downregulates *FOXP2* and upregulates *MDFIC*, decreasing protein levels of *FOXP2* and increasing those of *MDFIC*. Although each element deletion induces a significant decrease in the levels of mRNA expression of *FOXP2*, this reduction seems to be higher at the protein level for FOXP2-E^proximal^ cells. We speculate that some protein regulatory mechanisms would be involved but what exactly these mechanisms are is poorly understood. Our results, in line with recent massive data derived from next-generation DNA sequencing and proteomics, show a variation between mRNA and protein abundances. The observed variations between the FOXP2 protein amounts with its associated mRNA are probably due to the effect of post-transcriptional regulatory processes occurring after mRNA synthesis, translational and protein degradation regulations, controlling steady-state protein abundances [45]. We have demonstrated that the deletion of any one of the two enhancers, FOXP2-E^proximal^ or FOXP2-E^distal^, leads to significant changes of FOXP2 expression and, interestingly, also in the transcription of six well-known FOXP2 target genes. These data support the hypothesis that each of the deleted regions functions as an expression regulator in a specific regulatory network of *FOXP2* in a human cell line.

In the widely used HEK293 human cell line the expression of *FOXP2* and *MDFIC* is also altered, but in a different manner, suggesting that each enhancer might confer a tissue specific regulation to each gene. Based on the results derived from HEK293 and SK-N-MC cell lines, FOXP2-E^prox^ and FOXP2-E^distal^ elements appeared to affect FOXP2 and MDFIC promoters’ activity in a cell-specific manner. This possibility is also reinforced by our finding that in the SK-N-MC line the six FOXP2 targets we have assessed are all downregulated after the deletion of any of these two enhancers, which suggests that FOXP2 is acting as an activator of gene expression. In other cell lines, like SH-SY5Y, some of these target genes (e.g. *PM5*) have been found to be downregulated by FOXP2 [42, 43].

One of the key processes in the enhancer–promoter interaction is mediated by transcription factors and their binding to enhancer and/or promoter regions, but chromatin structure and transcribed enhancer RNAs (eRNAs) from active enhancers also regulate enhancer–promoter looping and the release of paused RNAPII, and it is well established that these elements vary between cell types [46]. SK-N-MC is a neuroblastoma cell line derived from the supraorbital area, whereas HEK293 cells are derived from the kidney, therefore, chromatin structure regulatory element accessibility, signalling pathways, gene expression profiles and many other cell components are likely to be quite different in the two cell types. The exact causes of enhancer-promoter cell-type activity differences and their specific regulation in human cells are poorly understood and remain yet to be fully elucidated [47]. That said, because the cell lines we have selected for our study are known to have a long track of chromosomal aberrations, an induced pluripotent stem (iPS)-based replication of our findings should help to confirm this cell-specific effect we have uncovered.

The effect on *FOXP2* and *MDFIC* gene expression regulation is coherent with previous studies reporting pairs of genes being governed by the same regulatory elements [48–50]. In some cases, these elements have proven to supercoil the DNA and to alter chromatin topology to facilitate or to hinder the assembly of the transcriptional machinery [51, 52]. Our results indicate that whereas each enhancer deletion induced a downregulation on FOXP2 expression, MDFIC expression was significantly upregulated. One possible explanation for this phenomenon is that the deletion of FOXP2-E^prox^ or FOXP2-E^distal^ regulatory element could have removed or interfered with an inhibitory regulation process of MDFIC expression, maybe through an indirect modification of the chromatin structure. These data suggest that removal of any one of these two intergenic enhancers likely disables intricate inter-enhancer interactions [53–56] which may be required to stabilize, within specific gene regulatory networks, gene-specific expression patterns in tissue-specific cell lines [47, 57].

*FOXP2* is a well-known gene, important for speech and language [6, 58]. Less is known about the role of *MDFIC* in cognitive development and disease. This gene encodes a MyoD family inhibitor domain containing protein that acts as an activator or repressor of transcription [59, 60]. Similarly to FOXP2, it interacts with LEF1, as part of beta-catenin regulation [61]. *MDFIC* is highly expressed in the cerebellum during human embryonic development and in the thalamus after birth (Human Brain Transcriptome http://hbatlas.org/). These two brain regions, interacting with others in a dopaminergic cortico-striato-thalamic loop, seem to play an important role in timing sensorimotor control, needed for auditory-motor language processing [1, 3, 10, 62].

Microdeletions affecting the *FOXP2-MDFIC* intergenic region have been reported to be associated with speech or cognitive impairment (Additional file 1: Figure S1). Most of these reported deletions encompass part of the coding region of *FOXP2*, and are confirmed or expected to involve some developmental language deficit. Interestingly, in one case entailing the deletion of FOXP2-E^distal^ only, coordination problems and learning disability have been reported (Additional file 1: Figure S1). Although this deletion encompasses the *MDFIC* gene, the reported phenotype recapitulates aspects of *FOXP2* mutation or deletion. Because we have proven that the deletion of FOXP2-E^distal^ downregulates *FOXP2* in a human neuronal cell line, we hypothesise that the speech and language deficits exhibited by our patient might result from the downregulation of *FOXP2* [23]. Nonetheless, because the breakpoint also separated *MDFIC* from FOXP2-E^proximal^, we also expect the expression of *MDFIC* to be upregulated in our proband’s brain cells. Additional studies should be conducted to prove or disprove our hypothesis. Interestingly, genomic rearrangements affecting topologically associating domains (TADs) can result in gene miss-expression and disease [50]. Genome engineering experiments aimed at deleting these two enhancers in other animal species could provide further support for their functionality. Bats seem a natural target, since they exhibit a learned vocal behaviour [63], these enhancer regions are conserved in most species (ENCODE data, Additional file 6: Figure S6), and gene delivery systems in the bat brain have been recently improved and tested with the *FoxP2* gene [64]. The study of a specific neuronal brain cell line differentiated from iPS cells, obtained from our proband and a relevant control to implement genome editing, could contribute as well to give additional support to our hypothesis.

## Conclusions

In summary, we expect that our findings, together with new available data about seed sequences of miRs in the 3’UTR region of *FOXP2* [22, 65]*,* contribute to a deeper understanding of how *FOXP2* is regulated, and ultimately, of its role in the development of the biological machinery underlying language.

## Additional files

Additional file 1: **Figure S1.** Genomic map of FOXP2 and MDFIC region. A. Chromosome 7 ideogram representation. Red box shows the region displayed below in Mb. B. Localization of FOXP2 (red track), MDFIC (yellow track), FOXP2-Eproximal and FOXP2-Edistal enhancers (green circles) and breakpoint locus (red asterisk). C. Deletions within the region of interest with a clinical significance as provided by DECIPHER (red tracks), showing the patients´ identification number (red). The genomic coordinates according to the hg19 (black), and the most relevant clinical features (blue). ID, intellectual disability. (JPG 71 kb)

Additional file 2: **Figure S2.** Indel spectrum determined by TIDE of the on-target sites compared with indel frequencies of the control sample. Each module represents the TIDE analysis of one sgRNA in a bulk cell population electroporated with each of the single-guide-Cas9 encoded plasmids. Each bar graph represents an indel event with an estimation of the percentage of the population exhibiting this particular event. Light-red bars represent the wild type control DNA sequence, bright-red bars represent significant indel events and black bars represent non-significant differences. *P*-values according to Pearson’s chi-squared test. Decomposition was limited to indels of size 0–10, hence larger indels could not be detected. R2 represent a quality measurement of the sequence reads. Indel % is represented at the top left site each module. (JPG 1103 kb)

Additional file 3: **Figure S3.** Indel spectrum determined by TIDE of the off-target sites compared with indel frequencies of the control sample. Each module represents the TIDE analysis of one sgRNA in a bulk cell population electroporated with each of the single-guide-Cas9 encoded plasmids. Each bar graph represents an indel event with an estimation of the percentage of the population exhibiting this particular event. On-target and potential off-target sequences are represented on top of each module and mismatched bases are shown in red. Light-red bars represent the wild type situation, bright-red bars represent significant indel events and black bars represent non-significant differences. P-values according to Pearson’s chi-squared test. Decomposition was limited to indels of size 0–10, hence larger indels could not be detected. R2 represent a quality measurement of the sequence reads. Indel % is represented at the top left site each module. (JPG 1041 kb)

Additional file 4: **Figure S4.** PCR analysis. Two oligos flanking the deleted regions were used to amplify the genomic DNA from several mutant representative HEK293 and SK-N-MC clones. Black triangles show the size of the PCR products. Black or white asterisks show respectively the clones harbouring a homozygous or heterozygous deletion included in this study. M: molecular weight marker, WT/WT: wild type, Δ/Δ: homozygous deletion, WT/Δ: heterozygous deletion. (JPG 1181 kb)

Additional file 5: **Figure S5.** RT-qPCR analysis of six HEK293 cell clones with FOXP2-Eproximal or FOXP2-Edistal deletions. Samples are normalized to the average FOXP2 (left) or MDFIC (right) signal between three HEK293 replicates transfected with the pLV-U6^#x^H1^#y^-C9G empty vector. Levels of expression of FOXP2 and MDFIC are represented by the fold change relative to that of empty vector control cell line, which were normalized to 1. WT/WT: wild type, Δ/Δ: homozygous deletion, WT/Δ: heterozygous deletion. (JPG 373 kb)

Additional file 6: **Figure S6.** Detailed view of an ENCODE UCSC genome-browser snapshot showing bar graphs with a detailed representation of the locations of FOXP2 and MDFIC genes, H3K27Ac and DNA clusters in human cell lines. The squared regions in black show the locations of FOXP2-Eproximal and FOXP2-Edistal. The red squared tracks show the alignment result between humans and bats. (JPG 761 kb)

Additional file 7: **Table S1.** Oligonucleotide and sgRNA sequences used in this study. (DOCX 15 kb)

## Abbreviations

CRISPR: Clustered regularly interspaced short palindromic repeats
eRNAs: enhancer RNAs
GFP: Green fluorescent protein
INDEL: Insertion or deletion
iPS: induced pluripotent stem (cells)
qRT-PCR: real-time quantitative RT-PCR
TAD: Topologically associating domain
UTR: Untranslated region
