# Supplementary figures and images for: Functional characterization of two enhancers located downstream FOXP2
Source: BMC Med Genet. 2019 May 2;20:65. doi: 10.1186/s12881-019-0810-2 (PMC6498672; doi:10.1186/s12881-019-0810-2)

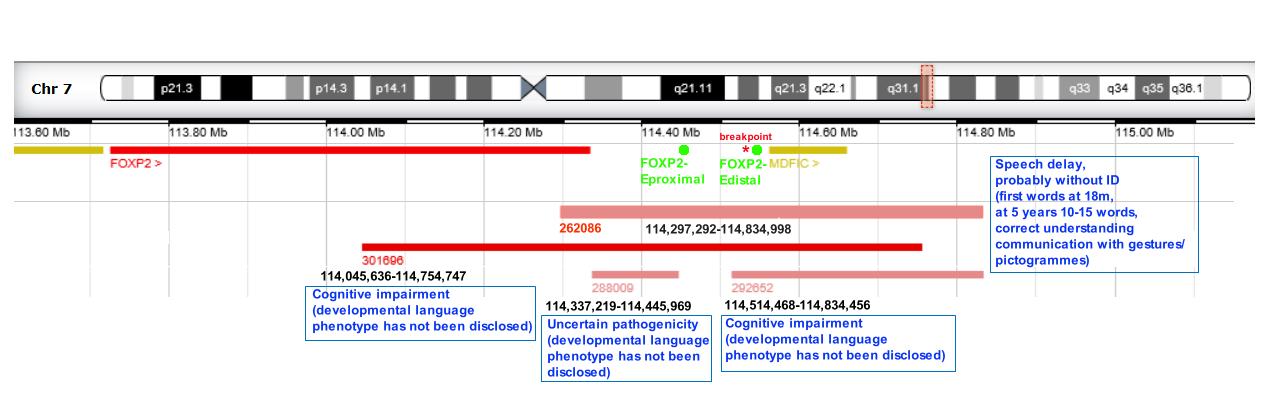

Supplement: Supplementary file 1 — Figure S1. Genomic map of FOXP2 and MDFIC region. A. Chromosome 7 ideogram representation. Red box shows the region displayed below in Mb. B. Localization of FOXP2 (red track), MDFIC (yellow track), FOXP2-Eproximal and FOXP2-Edistal enhancers (green circles) and breakpoint locus (red asterisk). C. Deletions within the region of interest with a clinical significance as provided by DECIPHER (red tracks), showing the patients´ identification number (red). The genomic coordinates according to the hg19 (black), and the most relevant clinical features (blue). ID, intellectual disability. (JPG 71 kb) [file 12881_2019_810_MOESM1_ESM.jpg]

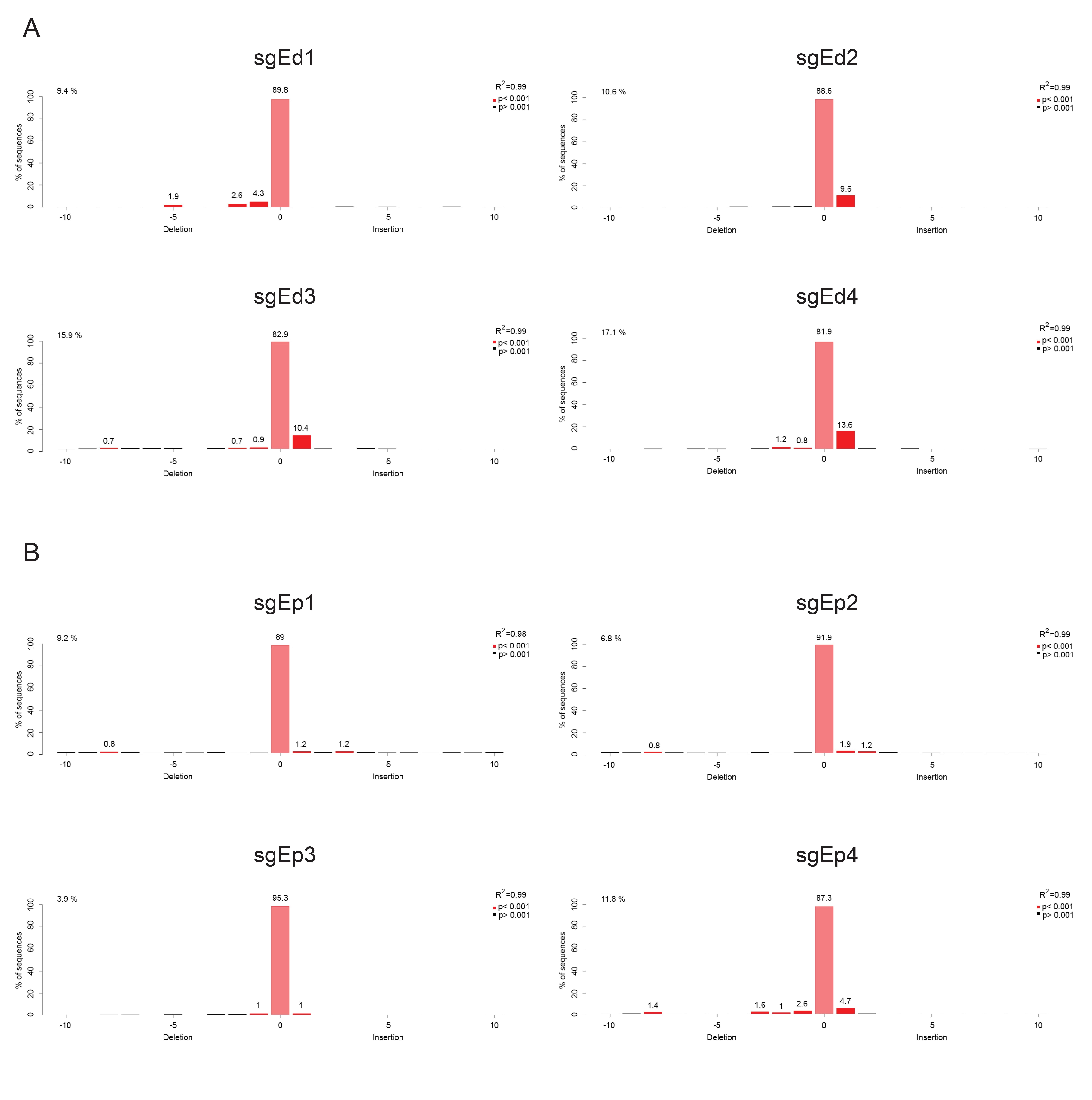

Supplement: Supplementary file 2 — Figure S2. Indel spectrum determined by TIDE of the on-target sites compared with indel frequencies of the control sample. Each module represents the TIDE analysis of one sgRNA in a bulk cell population electroporated with each of the single-guide-Cas9 encoded plasmids. Each bar graph represents an indel event with an estimation of the percentage of the population exhibiting this particular event. Light-red bars represent the wild type control DNA sequence, bright-red bars represent significant indel events and black bars represent non-significant differences. P-values according to Pearson’s chi-squared test. Decomposition was limited to indels of size 0–10, hence larger indels could not be detected. R2 represent a quality measurement of the sequence reads. Indel % is represented at the top left site each module. (JPG 1103 kb) [file 12881_2019_810_MOESM2_ESM.jpg]

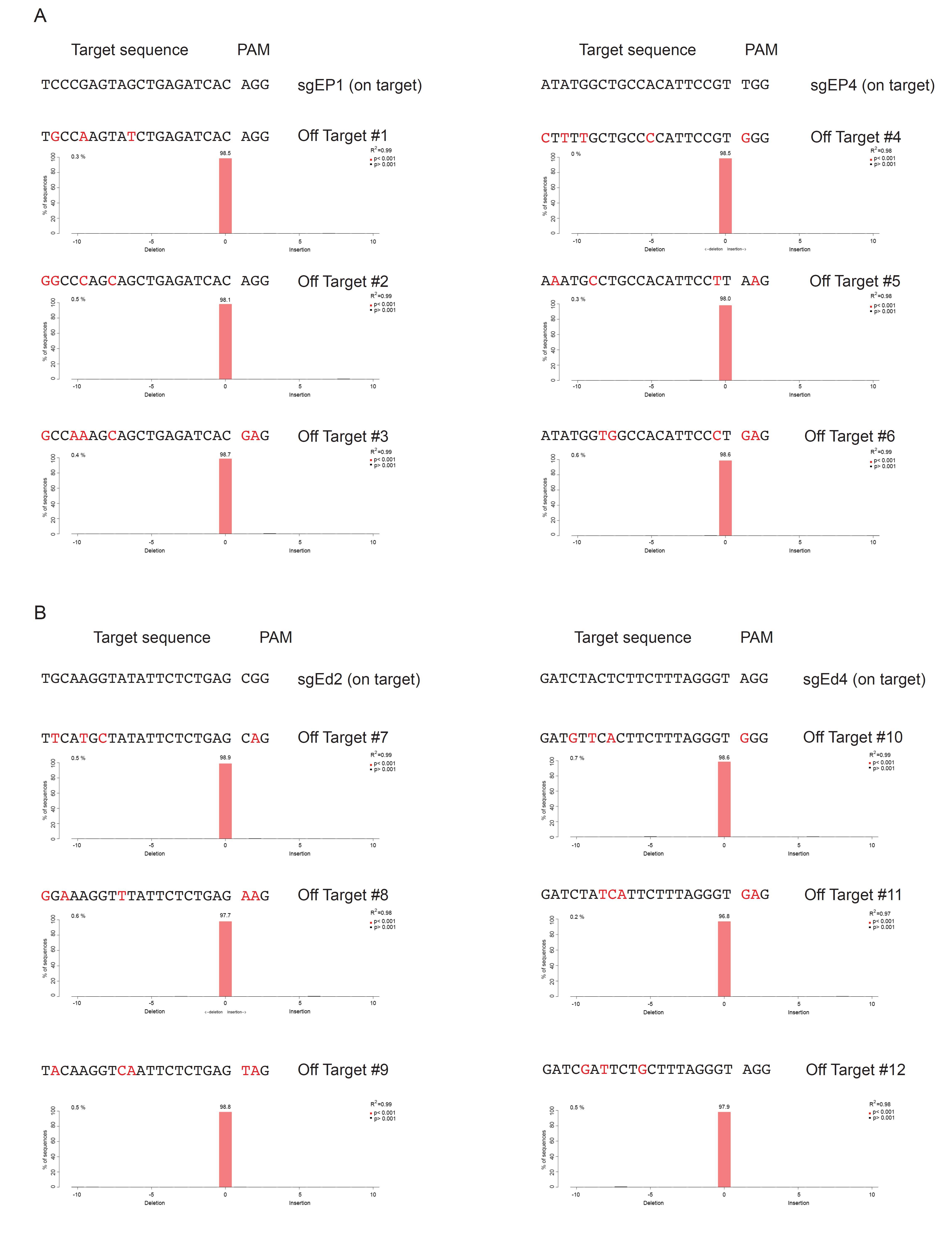

Supplement: Supplementary file 3 — Figure S3. Indel spectrum determined by TIDE of the off-target sites compared with indel frequencies of the control sample. Each module represents the TIDE analysis of one sgRNA in a bulk cell population electroporated with each of the single-guide-Cas9 encoded plasmids. Each bar graph represents an indel event with an estimation of the percentage of the population exhibiting this particular event. On-target and potential off-target sequences are represented on top of each module and mismatched bases are shown in red. Light-red bars represent the wild type situation, bright-red bars represent significant indel events and black bars represent non-significant differences. P-values according to Pearson’s chi-squared test. Decomposition was limited to indels of size 0–10, hence larger indels could not be detected. R2 represent a quality measurement of the sequence reads. Indel % is represented at the top left site each module. (JPG 1041 kb) [file 12881_2019_810_MOESM3_ESM.jpg]

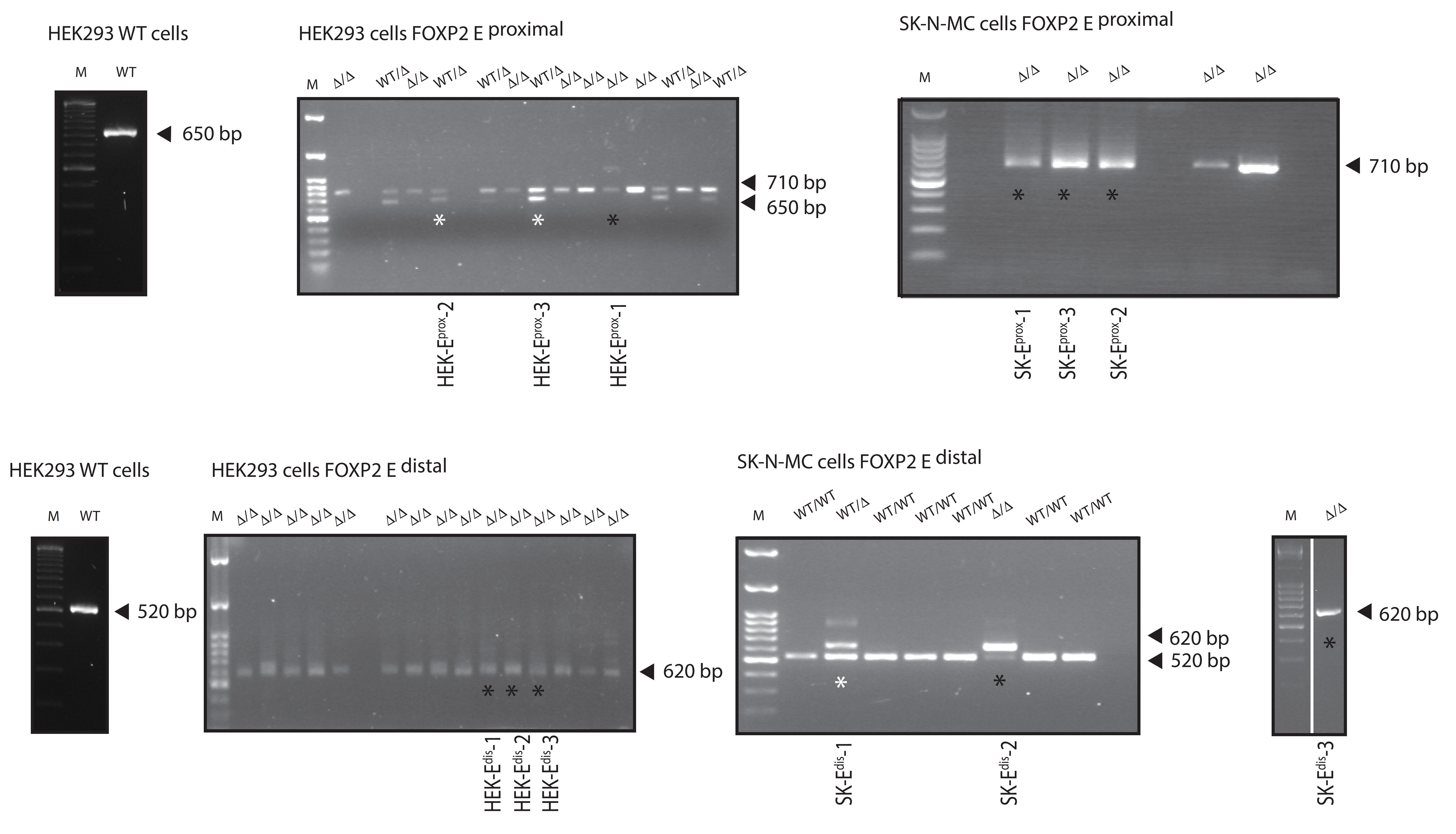

Supplement: Supplementary file 4 — Figure S4. PCR analysis. Two oligos flanking the deleted regions were used to amplify the genomic DNA from several mutant representative HEK293 and SK-N-MC clones. Black triangles show the size of the PCR products. Black or white asterisks show respectively the clones harbouring a homozygous or heterozygous deletion included in this study. M: molecular weight marker, WT/WT: wild type, Δ/Δ: homozygous deletion, WT/Δ: heterozygous deletion. (JPG 1181 kb) [file 12881_2019_810_MOESM4_ESM.jpg]

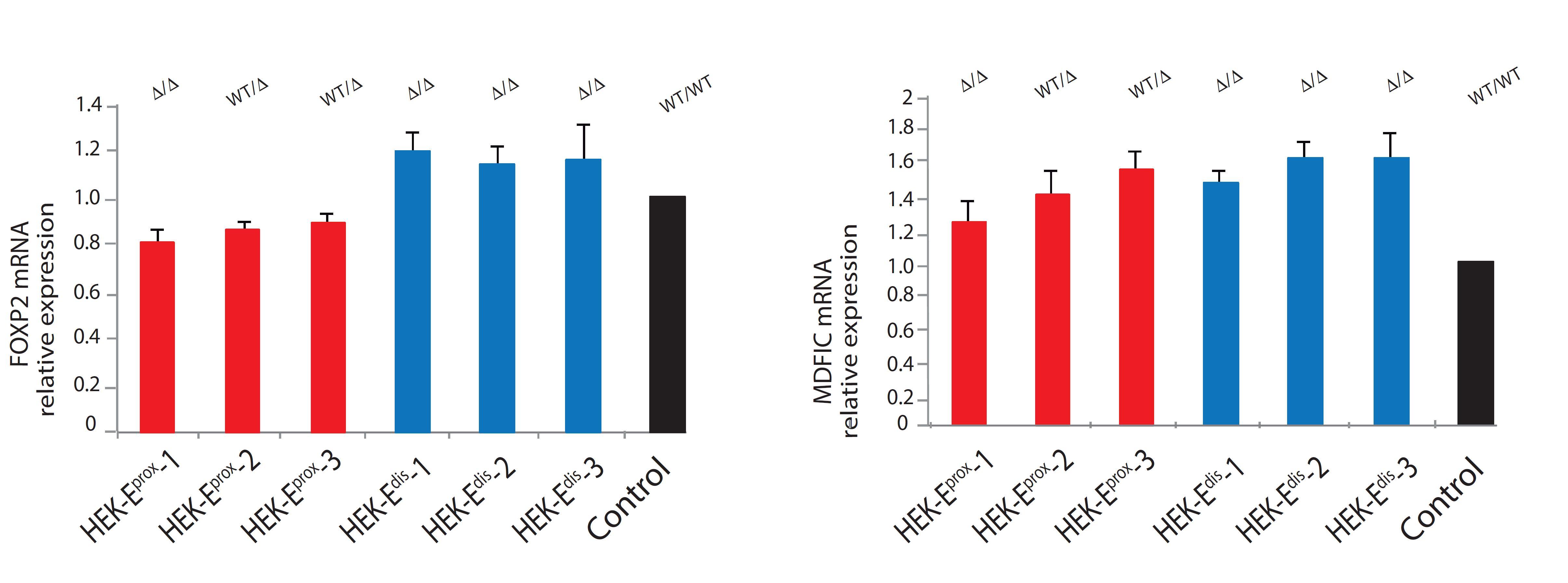

Supplement: Supplementary file 5 — Figure S5. RT-qPCR analysis of six HEK293 cell clones with FOXP2-Eproximal or FOXP2-Edistal deletions. Samples are normalized to the average FOXP2 (left) or MDFIC (right) signal between three HEK293 replicates transfected with the pLV-U6#xH1#y-C9G empty vector. Levels of expression of FOXP2 and MDFIC are represented by the fold change relative to that of empty vector control cell line, which were normalized to 1. WT/WT: wild type, Δ/Δ: homozygous deletion, WT/Δ: heterozygous deletion. (JPG 373 kb) [file 12881_2019_810_MOESM5_ESM.jpg]

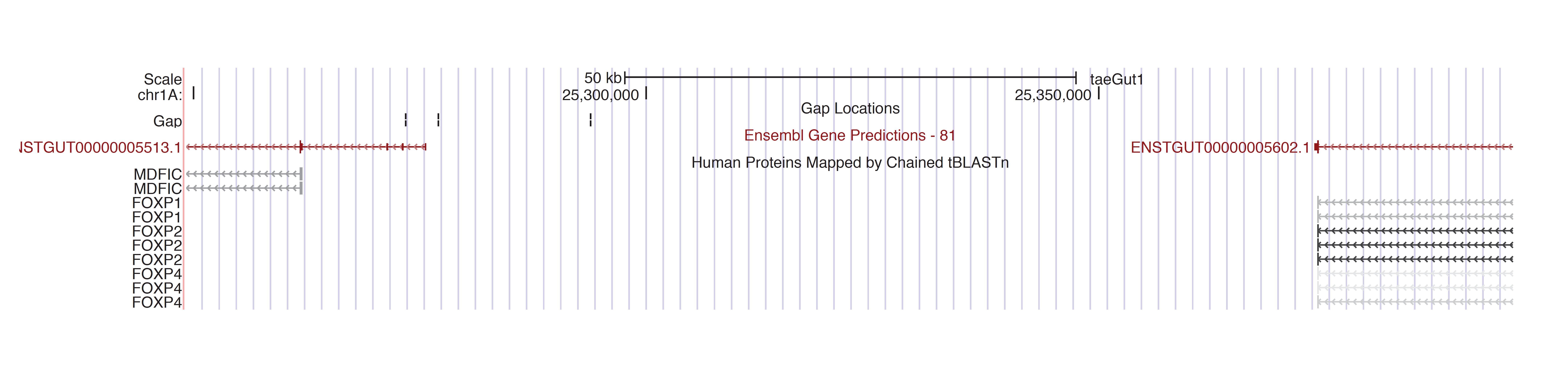

Supplement: Supplementary file 6 — Figure S6. Detailed view of an ENCODE UCSC genome-browser snapshot showing bar graphs with a detailed representation of the locations of FOXP2 and MDFIC genes, H3K27Ac and DNA clusters in human cell lines. The squared regions in black show the locations of FOXP2-Eproximal and FOXP2-Edistal. The red squared tracks show the alignment result between humans and bats. (JPG 761 kb) [file 12881_2019_810_MOESM6_ESM.jpg]
