# Supplementary material for: Functional characterization of two enhancers located downstream FOXP2
Source: BMC Med Genet. 2019 May 2;20:65. doi: 10.1186/s12881-019-0810-2 (PMC6498672; doi:10.1186/s12881-019-0810-2)
Supplement: Supplementary file 7 — Table S1. Oligonucleotide and sgRNA sequences used in this study. (DOCX 15 kb) [file 12881_2019_810_MOESM7_ESM.docx]

**sgRNA sequences (IDT gblocks)**

**sgRNA_FOXP2_Edistal_1_5 (sgEd#1):**

ccataACGCGTTGTACACGAACGCTGACGTCATCAACCCGCTCCAAGGAATCGCGGGCCCAGTGTCACTAGGCGGGAACACCCAGCGCGCGTGCGCCCTGGCAGGAAGATGGCTGTGAGGGACAGGGGAGTGGCGCCCTGCAATATTTGCATGTCGCTATGTGTTCTGGGAAATCACCATAAACGTGAAATGTCTTTGGATTTGGGAATCTTATAAGTTCTGTATGAGACCACTCTTTCCC**G**cacacccagcaaaatacatGTTTTAGAGCTATGCTGGAAACAGCATAGCAAGTTAAAATAAGGCTAGTCCGTTATCAACTTGAAAAAGTGGCACCGAGTCGGTGCTTTTTTACTAGTcgcta

**sgRNA_FOXP2_Edistal_2_5 (sgEd#2):**

ccataACGCGTTGTACACGAACGCTGACGTCATCAACCCGCTCCAAGGAATCGCGGGCCCAGTGTCACTAGGCGGGAACACCCAGCGCGCGTGCGCCCTGGCAGGAAGATGGCTGTGAGGGACAGGGGAGTGGCGCCCTGCAATATTTGCATGTCGCTATGTGTTCTGGGAAATCACCATAAACGTGAAATGTCTTTGGATTTGGGAATCTTATAAGTTCTGTATGAGACCACTCTTTCCC**G**gcaaggtatattctctgagGTTTTAGAGCTATGCTGGAAACAGCATAGCAAGTTAAAATAAGGCTAGTCCGTTATCAACTTGAAAAAGTGGCACCGAGTCGGTGCTTTTTTACTAGTcgcta

**sgRNA_FOXP2_Edistal_1_3 (sgEd#3):**

ccataCAATTGGGGCAGGAAGAGGGCCTATTTCCCATGATTCCTTCATATTTGCATATACGATACAAGGCTGTTAGAGAGATAATTAGAATTAATTTGACTGTAAACACAAAGATATTAGTACAAAATACGTGACGTAGAAAGTAATAATTTCTTGGGTAGTTTGCAGTTTTAAAATTATGTTTTAAAATGGACTATCATGTACACTTACCGTAACTTGAAAGTATTTCGATTTCTTGGCTTTATATATCTTGTGGAAAGGACGAGGTACC**G**atctactcttctttagggtGTTTTAGAGCTATGCTGGAAACAGCATAGCAAGTTAAAATAAGGCTAGTCCGTTATCAACTTGAAAAAGTGGCACCGAGTCGGTGCTTTTTTACGCGTACTAGTcgcta

**sgRNA_FOXP2_Edistal_2_3 (sgEd#4):**

ccataCAATTGGGGCAGGAAGAGGGCCTATTTCCCATGATTCCTTCATATTTGCATATACGATACAAGGCTGTTAGAGAGATAATTAGAATTAATTTGACTGTAAACACAAAGATATTAGTACAAAATACGTGACGTAGAAAGTAATAATTTCTTGGGTAGTTTGCAGTTTTAAAATTATGTTTTAAAATGGACTATCATGTACACTTACCGTAACTTGAAAGTATTTCGATTTCTTGGCTTTATATATCTTGTGGAAAGGACGAGGTACC**G**gaagagtagatcgcatgagGTTTTAGAGCTATGCTGGAAACAGCATAGCAAGTTAAAATAAGGCTAGTCCGTTATCAACTTGAAAAAGTGGCACCGAGTCGGTGCTTTTTTACGCGTACTAGTcgcta

**sgRNA_FOXP2_Eproximal_1_5 (sgEp#1):**

ccataACGCGTTGTACACGAACGCTGACGTCATCAACCCGCTCCAAGGAATCGCGGGCCCAGTGTCACTAGGCGGGAACACCCAGCGCGCGTGCGCCCTGGCAGGAAGATGGCTGTGAGGGACAGGGGAGTGGCGCCCTGCAATATTTGCATGTCGCTATGTGTTCTGGGAAATCACCATAAACGTGAAATGTCTTTGGATTTGGGAATCTTATAAGTTCTGTATGAGACCACTCTTTCCC**G**gtgatctcagctactcgggGTTTTAGAGCTATGCTGGAAACAGCATAGCAAGTTAAAATAAGGCTAGTCCGTTATCAACTTGAAAAAGTGGCACCGAGTCGGTGCTTTTTTACTAGTcgcta

**sgRNA_FOXP2_Eproximal_2_5 (sgEp#2):**

ccataACGCGTTGTACACGAACGCTGACGTCATCAACCCGCTCCAAGGAATCGCGGGCCCAGTGTCACTAGGCGGGAACACCCAGCGCGCGTGCGCCCTGGCAGGAAGATGGCTGTGAGGGACAGGGGAGTGGCGCCCTGCAATATTTGCATGTCGCTATGTGTTCTGGGAAATCACCATAAACGTGAAATGTCTTTGGATTTGGGAATCTTATAAGTTCTGTATGAGACCACTCTTTCCC**G**ctcgaacttctgacctcagGTTTTAGAGCTATGCTGGAAACAGCATAGCAAGTTAAAATAAGGCTAGTCCGTTATCAACTTGAAAAAGTGGCACCGAGTCGGTGCTTTTTTACTAGTcgcta

**sgRNA_FOXP2_Eproximal_1_3 (sgEp#3):**

ccataCAATTGGGGCAGGAAGAGGGCCTATTTCCCATGATTCCTTCATATTTGCATATACGATACAAGGCTGTTAGAGAGATAATTAGAATTAATTTGACTGTAAACACAAAGATATTAGTACAAAATACGTGACGTAGAAAGTAATAATTTCTTGGGTAGTTTGCAGTTTTAAAATTATGTTTTAAAATGGACTATCATGTACACTTACCGTAACTTGAAAGTATTTCGATTTCTTGGCTTTATATATCTTGTGGAAAGGACGAGGTACC**G**ctgtaataagatagcagggGTTTTAGAGCTATGCTGGAAACAGCATAGCAAGTTAAAATAAGGCTAGTCCGTTATCAACTTGAAAAAGTGGCACCGAGTCGGTGCTTTTTTACGCGTACTAGTcgcta

**sgRNA_FOXP2_Eproximal_2_3 (sgEp#4):**

ccataCAATTGGGGCAGGAAGAGGGCCTATTTCCCATGATTCCTTCATATTTGCATATACGATACAAGGCTGTTAGAGAGATAATTAGAATTAATTTGACTGTAAACACAAAGATATTAGTACAAAATACGTGACGTAGAAAGTAATAATTTCTTGGGTAGTTTGCAGTTTTAAAATTATGTTTTAAAATGGACTATCATGTACACTTACCGTAACTTGAAAGTATTTCGATTTCTTGGCTTTATATATCTTGTGGAAAGGACGAGGTACC**G**tatggctgccacattccgtGTTTTAGAGCTATGCTGGAAACAGCATAGCAAGTTAAAATAAGGCTAGTCCGTTATCAACTTGAAAAAGTGGCACCGAGTCGGTGCTTTTTTACGCGTACTAGTcgcta

**Primers qPCR**

> qFoxP2_Fw

GCAGCAGAGATGGAAGATCA

> qFoxP2_Rv

AGTTGTCTTGCTGCCTGGAG

cDNA amplicon size:103 Estimated genomic amplicon size:108040

> qMDFIC_Fw

GTCCATTTGGGGAAATCCTT

> qMDFIC_Rv

CATTGCTCAGACCTGTGTGG

cDNA amplicon size:140 Estimated genomic amplicon size:37248

**Primers Surveyor & deletion detection**

sMDFIC Fw2

TGATCTCAGTGCAGGCAAA

sMDFIC Rv2

GTTGGACTAAGGTGCCAGTT

2314pb (deletion FOXP2 Distal)

sMDFIC Fw

TACTGTTTCATGGATGCTGACT

sMDFIC Rv

CCTTTGGCCACAGACTGAA

sFOXP2 Fw

GGGATAGCACTGGGAGAAATAC

sFOXP2 Rv

GCGGTGGCTCATTTCTGTA

6264 pb (deletion FOXP2 Proximal)

sFOXP2 Fw2

TTCTGCACCTTGGGTTAGG

sFOXP2 Rv2

AGGGTTGATTGATTGCCAGAG
